# UniLectin3D, a database of carbohydrate binding proteins with curated information on 3D structures and interacting ligands

**DOI:** 10.1093/nar/gky832

**Published:** 2018-09-17

**Authors:** François Bonnardel, Julien Mariethoz, Sebastian Salentin, Xavier Robin, Michael Schroeder, Serge Perez, Frédérique Lisacek, Anne Imberty

**Affiliations:** 1 Univ. Grenoble Alpes, CNRS, CERMAV, 38000 Grenoble, France; 2 Proteome Informatics Group, SIB Swiss Institute of Bioinformatics, CH-1227 Geneva, Switzerland; 3 Department of Computer Science, University of Geneva, Route de Drize 7, CH-1227 Geneva, Switzerland; 4 Biotechnology Center (BIOTEC), TU Dresden, Tatzberg 47–49, 01307 Dresden, Germany; 5 Biozentrum, University of Basel, Klingelbergstrasse 50–70, CH-4056 Basel, Switzerland; 6 Computational Structural Biology Group, SIB Swiss Institute of Bioinformatics, CH-4056 Basel, Switzerland; 7 Univ. Grenoble Alpes, CNRS, DPM, 38000 Grenoble, France; 8 Section of Biology, University of Geneva, CH-1205 Geneva, Switzerland

## Abstract

Lectins, and related receptors such as adhesins and toxins, are glycan-binding proteins from all origins that decipher the glycocode, i.e. the structural information encoded in the conformation of complex carbohydrates present on the surface of all cells. Lectins are still poorly classified and annotated, but since their functions are based on ligand recognition, their 3D-structures provide a solid foundation for characterization. UniLectin3D is a curated database that classifies lectins on origin and fold, with cross-links to literature, other databases in glycosciences and functional data such as known specificity. The database provides detailed information on lectins, their bound glycan ligands, and features their interactions using the Protein–Ligand Interaction Profiler (PLIP) server. Special care was devoted to the description of the bound glycan ligands with the use of simple graphical representation and numerical format for cross-linking to other databases in glycoscience. We conceived the design of the database architecture and the navigation tools to account for all organisms, as well as to search for oligosaccharide epitopes complexed within specified binding sites. UniLectin3D is accessible at https://www.unilectin.eu/unilectin3D.

## INTRODUCTION

Glycoscience is the discipline that studies carbohydrate-based biomolecules, including monosaccharides, oligosaccharides, polysaccharides and conjugates containing carbohydrate moieties. The roles of such sugars (also referred to as glycans or glycoconjugates) are manifold and they underline various medical, biochemical and biotechnological applications (1). All eukaryotic cells are covered by a dense layer of glyco-conjugates and the cell walls of bacteria are made of various polysaccharides. The variety of monosaccharides and the different topologies of the glycosidic bonds assembling oligosaccharides into branched structures define the range of conformations adopted by glycans at the surface of cells, resulting in the presentation of glyco-epitopes (2). These epitopes vary from one tissue (or one cell) to the others and the variation of glycosylation is related to a wide range of pathologies. Deciphering the glycocode, i.e. the information carried in the 3D-structure of glycans, is therefore of general interest.

The complexity of cell surface glycan structures and the corresponding sophisticated biosynthetic machinery required to assemble them are matched by the variety of protein receptors able to recognize them. Among them, lectins are carbohydrate-binding proteins that decipher the glycocode (3,4). Lectins are present in all living organisms, from virus to plants or mammals (3,5). They are involved in cell–cell interaction and signaling and play a role in innate immunity through their ability to discriminate between self and non-self (6). They are key elements in many infectious processes and therefore are the targets for the development of novel classes of anti-infectious compounds (7). Lectins can be engineered, providing new tools for biomarkers and vectorization (8). They are also used in glycomics studies notably with the recent development of lectin arrays (9,10). Until recently, carbohydrate structures were poorly annotated in the protein data bank, but initiatives such as pdb-care (11) and Privateer (12) now provide tools for controlling stereochemistry and quality of carbohydrate moieties in 3D-structures.

The structural knowledge of the binding sites and of their interactions with ligands is a prerequisite for the design of high-affinity glycomimetic ligands. The fine description of the network of hydrogen bonds, van der Waals contacts, ionic bonds, but also of the bridging water molecules and the bivalent cations is imperative. Further insight into the oligomeric state is equally important: lectins generally exhibit several carbohydrate binding sites, resulting in strong avidity to the dense structure of glycans at the surface of cells. The design of multivalent glyco-compounds able to fit with the lectin topology allows for the synthesis of highly specific ligands (13). While active carbohydrate enzymes are well covered in the CAZy database (http://www.cazy.org/) (14), lectins are still poorly characterized and cross-referenced. A collection of lectin structures is available in the Glyco3D portal (http://glyco3d.cermav.cnrs.fr) that provides a broad range of structural information for the glycosciences (15). Two other databases are maintained and accessible: the Lectin Frontier Database (LfDB: https://acgg.asia/lfdb2/) provides information on the specificity of 400 lectins (16), and LectinDB (http://proline.physics.iisc.ernet.in/lectindb/) records information mainly about sequences of plant lectins (17). However, no database provides full interconnection between structural information on lectin and other bioinformatics resources related to lectins, ligands and function.

The UniLectin3D project was launched to address the urgent need for bridging the gap between glycoscience and other more prominent fields of molecular biology, which has been emphasized in many recent reviews and viewpoints (18–20). It is now increasingly accepted that this issue can be tackled throughout the expansion of glyco(bio)informatics. As a result, international consortia have recently invested significant effort in developing bioinformatics in glycomics such as GlyTouCan (https://glytoucan.org/) the universal glycan structure repository (21) or Glycomics@ExPASy (https://www.expasy.org/glycomics) the glycomics and glycoproteomics portal (22). The latter hosts SugarbindDB (https://sugarbind.expasy.org/) that provides information on known carbohydrate sequences and their receptors from bacteria, toxins and viruses (23).

The content and use of UniLectin3D is described below. It is centered on 3D data, using PDB information, with associate protein sequence features and appropriate curation of the glycan topology. It provides a family-based classification and cross-links to specialized glyco-related databases. In particular, each carbohydrate ligand can be seen as part of the full carbohydrate structures. Finally, the 3D visualization of contacts between the lectin and the ligand is visualized via the Protein–Ligand Interaction Profiler (PLIP) server (24).

## DATA SOURCE AND DATABASE CONSTRUCTION

UniLectin3D is available at https://www.unilectin.eu/unilectin3D. It is developed with PHP version 7, Bootstrap version 3 and MySQL database version 5.6. The interface is compatible with all devices and browsers. The pages are dynamically generated to match the research criteria selected by the user in the query window. Interactive graphics are developed in JavaScript based on D3JS libraries version 3. A tutorial can be accessed on the first page.

The UniLectin3D database is populated with (i) the lectins referenced in the Glyco3D database until 2016, (ii) manually filtered PDB structures based on the weekly release and (iii) PDB structures containing a carbohydrate processed with PLIP to filter lectins from glycoproteins, glycan transporters or cazymes. Based on manual filtering, lectin structures are classified by Origin, Class and Family. The Class divides lectins into large groups based on structures. The Family divides lectins mostly by species. This classification is further refined according to the type of protein fold and to the nature of the linked glycans. The glycan description, i.e. monosaccharide content and condensed IUPAC nomenclature (http://www.sbcs.qmul.ac.uk/iupac/2carb/38.html), is entered by manual curation with correction of possible errors in the PDB nomenclature.

**Figure 1.**
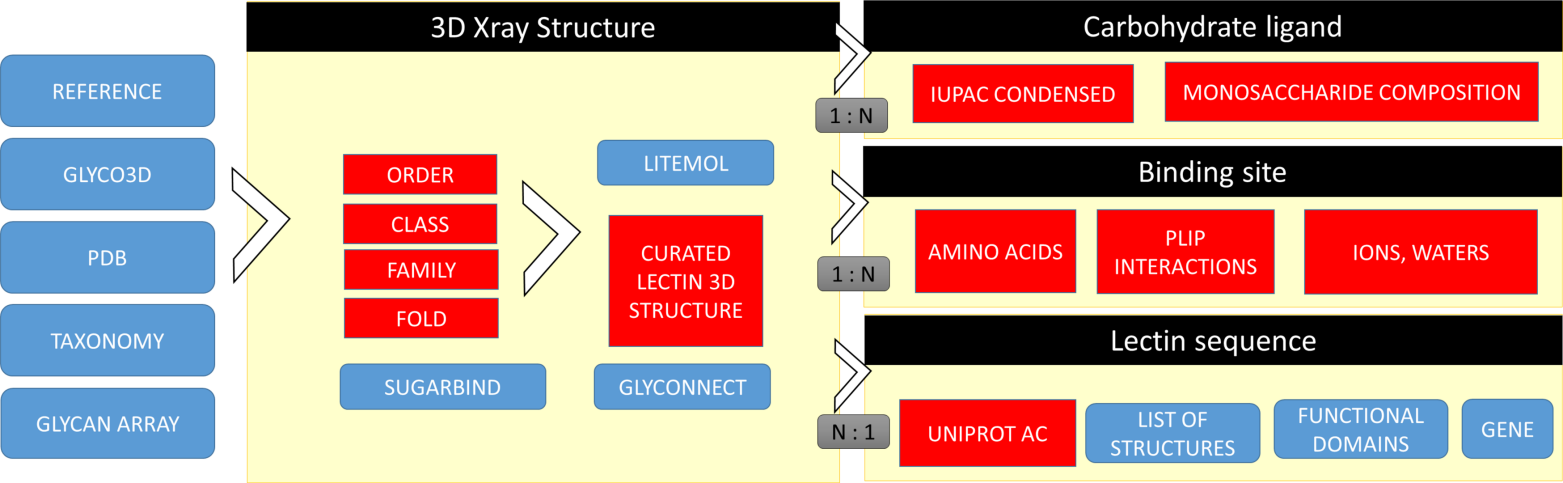
Flow chart illustrating the construction process and the information that users can obtain from the UniLectin3D database. Red boxes and blue boxes denote manually curated information and external data sources, respectively. Gray boxes represent relations between objects. (1:*N*) indicates that one X-ray structure is related to one or more ligands (and binding sites), (*N*:1) indicates that several X-ray structures may be related to one protein sequence.

UniLectin3D cross-references several other databases (Table 1). It links to the glyco-related SugarBindDB, GlyConnect (https://glyconnect.expasy.org) and GlyTouCan (https://glytoucan.org/) through the shared IUPAC sequences of glycans. A control of the quality of the glycan structure and stereochemistry is provided by a link to PDB-care (www.glycosciences.de/tools/pdb-care/). Since SugarBindDB also contains protein data, other links from UniLectin3D are made via UniProt accession numbers (25). Information about the specificity of lectins is provided through links to the Consortium for Functional Glycomics (http://www.functionalglycomics.org). The taxonomy identifier and PubMed reference links are based on the information collected in the corresponding PDB entries. The general flowchart of UniLecin is displayed in Figure 1. A unique lectin, as referenced by a UniProt accession number, may be associated with multiple PDB structures. Lectins stored in the curated section of UniLectin3D have at least one referenced structure.

**Table 1.** List of databases and websites that are cross-referenced or used in UniLectin3D

Database name	URL	Information	Reference
PDBe	https://www.ebi.ac.uk/pdbe/	3D structure and structural information	(41)
RCSB	https://www.rcsb.org/	3D structure and structural information	(36)
SWISS-MODEL templates	https://swissmodel.expasy.org/templates/	Information on quaternary structure	(39)
SugarBind	https://sugarbind.expasy.org/	Specificity of lectins from pathogens	(23)
GlyConnect	https://glyconnect.expasy.org/	Information on glycoproteins	
GlyTouCan	https://glytoucan.org/	International glycan structure repository	(21)
Glyco3D	http://glyco3d.cermav.cnrs.fr	3D structure and information of lectins	(15)
SNFG	https://www.ncbi.nlm.nih.gov/glycans/snfg.html	Symbol nomenclature for glycans	(37)
PubMed	http://www.ncbi.nlm.nih.gov/pubmed	Bibliographic information	
pdb-care	http://www.glycosciences.de/tools/pdb-care/	Structural check of carbohydrates	(11)
Taxonomy	http://www.ncbi.nlm.nih.gov/taxonomy	Organism taxonomy	
UniProtKB	http://www.uniprot.org	Lectin functional annotation	(25)
CFG	http://www.functionalglycomics.org	Experimental data of lectins specificity	
PLIP	http://plip.biotec.tu-dresden.de	Identification of protein–ligand interactions	(24)
LiteMol	https://www.litemol.org/	3D visualization of lectin structure	(34)
NGL	http://nglviewer.org/	Molecular graphics	(40)
ProtVista	http://ebi-uniprot.github.io/ProtVista/	Visualization and annotation of sequences	(27)

## OVERVIEW OF LECTIN DATA

**Figure 2.**
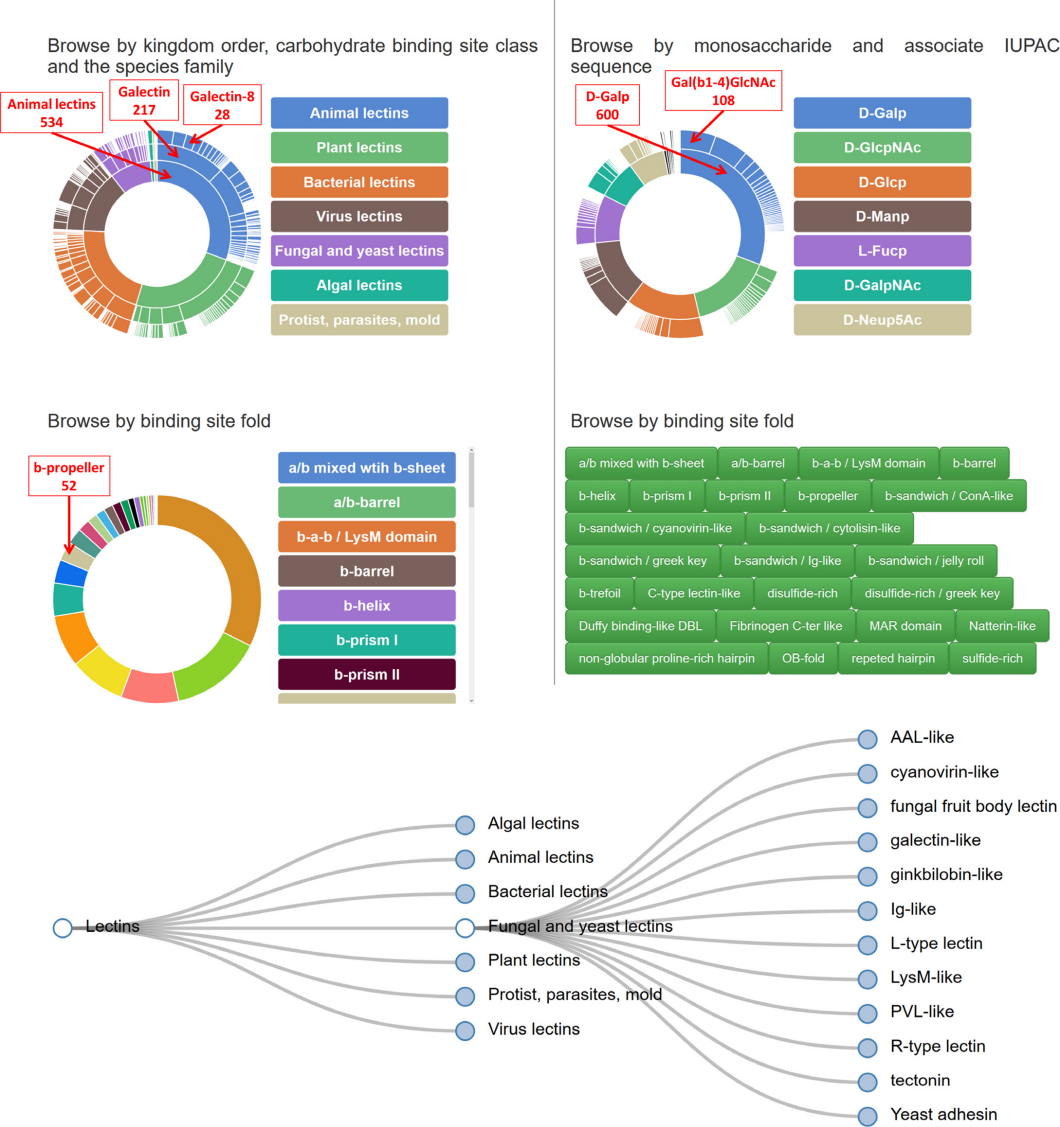
Different modes for browsing the database and obtaining statistics. The three sunburst charts provide access to classification, glycan ligand sequence and structural fold, respectively. The red rectangles give examples of statistics obtained when passing the mouse over the area. Each zone can be clicked to open the corresponding advanced search window. A full clickable list of folds is also provided. The hierarchical tree can be expanded by clicking on the blue circle and at each level, an advanced search window can be opened by clicking on the label.

The UniLectin3D database contains 1740 lectin structures that have been manually curated; this corresponds to 428 different lectin sequences (as of 29 August 2018). Bibliographic entries cover 765 published articles describing at least one structure. The first classification level, referred to as Origin, separates the lectins into the main kingdoms of the living world. The second level orders the lectins according to the protein fold into 75 classes. The third level separates the lectins according to their species in 309 families. Some statistics can be obtained from the sunbursts displayed on the home page (Figure 2).

One-third (534) of the lectin structures are from animal origin. The most populated class of animal lectins are the galectins; these proteins bind specifically to β-galactoside containing glycans. The second most populated class is the C-type lectins that contain calcium in their binding sites. Plant lectins, which were the first to be studied, represent 24% (416) of the content of the database. They comprise the very large and diverse family of L-type lectins (previously described as legume lectins) and the jacalin-like lectins with subfamilies specific for mannose and galactose. There is a growing number of 3D structures of lectins that are reported to occur in bacteria 21% (368), viruses 13.6% (239) and fungi 9% (157). The number of structures of lectins from algae, protists, parasites and molds is growing slowly reflecting the pace of elucidation of their biological functions.

The lectin fold is defined by the relative spatial arrangement of secondary structure elements. It characterizes the multiplicity of binding to glycans and is directly linked to the ability of lectins to bind and cross-link glycan-containing molecules in a multivalent fashion. The current trend in the PDB shows a strong predominance of β-sheet structural motifs. Two such β-sheets can assemble to form a β-sandwich, an architecture commonly found in more than half of the entries. These β-sandwiches exhibit dissimilarities and the location of binding sites shows many variations. For example, the immunoglobulin-like fold of animal sialo-adhesins strikingly differs from the jelly-roll fold of legume lectins. Lectins adopt mainly the ConA-like β-sandwich fold 32% (563), 14% (249) are β-sandwich jelly roll, 9% (156) are C-type lectin-like, 8% (151) are β-sandwich greek key. About 8% (140) are β-trefoil and 5% (90) are β-prism1.

The different types of glycans recognized by the lectins reflect the diversity of interactions at the cellular level. They can provide insight into those occurring between hosts and pathogens. Among the 1740 3D structures, 1120 occur as complexed with glycans. The most commonly observed monosaccharides are as follows: Galactose (Gal) in 600 different structures, i.e. 53% of the total of them; N-acetyl glucosamine (GlcNAc) 27% (302), glucose (Glc) 24% (275), mannose (Man) 22% (251), fucose (Fuc) 16%(179), N-acetyl galactosamine (GalNAc) 13% (145), sialic acid (Neu5Ac) 12% (134), but rarer sugars are also observed in complexes with lectin (Rhamnose, Arabinose …). The ligands occur as monosaccharides, but also as oligosaccharides or glycoconjugates with information about glycosidic linkages. The set of distinct glycan ligands amounts to 188.

In order to facilitate navigation, an interactive hierarchical tree shows the current lectin classification along with its division in subcategories (Figure 2). The advanced search of the application can be reached from any branch. Activating the search button in the corresponding window prompts the display of all the recorded lectin structures. Alternatively, each branch node of the tree can be clicked to access subcategories. In this representation, the occurrence of smaller categories and or nodes are not hindered. In this way, less investigated lectin category can be brought out.

## SEARCHING FOR SPECIFIC LECTINS

The variety of structures and binding sites can be explored by the sunburst viewers and the tree viewer (Figure 2). A simple search box is available on the UniLectin3D home page and allows searching by keywords, PDB or UniProt accession numbers, fragments of glycan sequences or textual fragments of the title of a publication. The advanced search offers a selection of criteria. Lectin can be searched by structure or by sequence family with the support of drop-down lists. The classification of lectins (Origin, Class and Family) also provides several search criteria. Other criteria pertain to the nature of the fold and taxonomic details of the lectins. Keywords from the title in a reference article can also be searched. A unique feature is the search of monosaccharides or fragments of glycan ligands that will output lectins interacting with a given carbohydrate. Finally, a cutoff on the resolution (Å) of the X-ray structure can be used as a filter for selecting of high-quality data.

**Figure 3.**
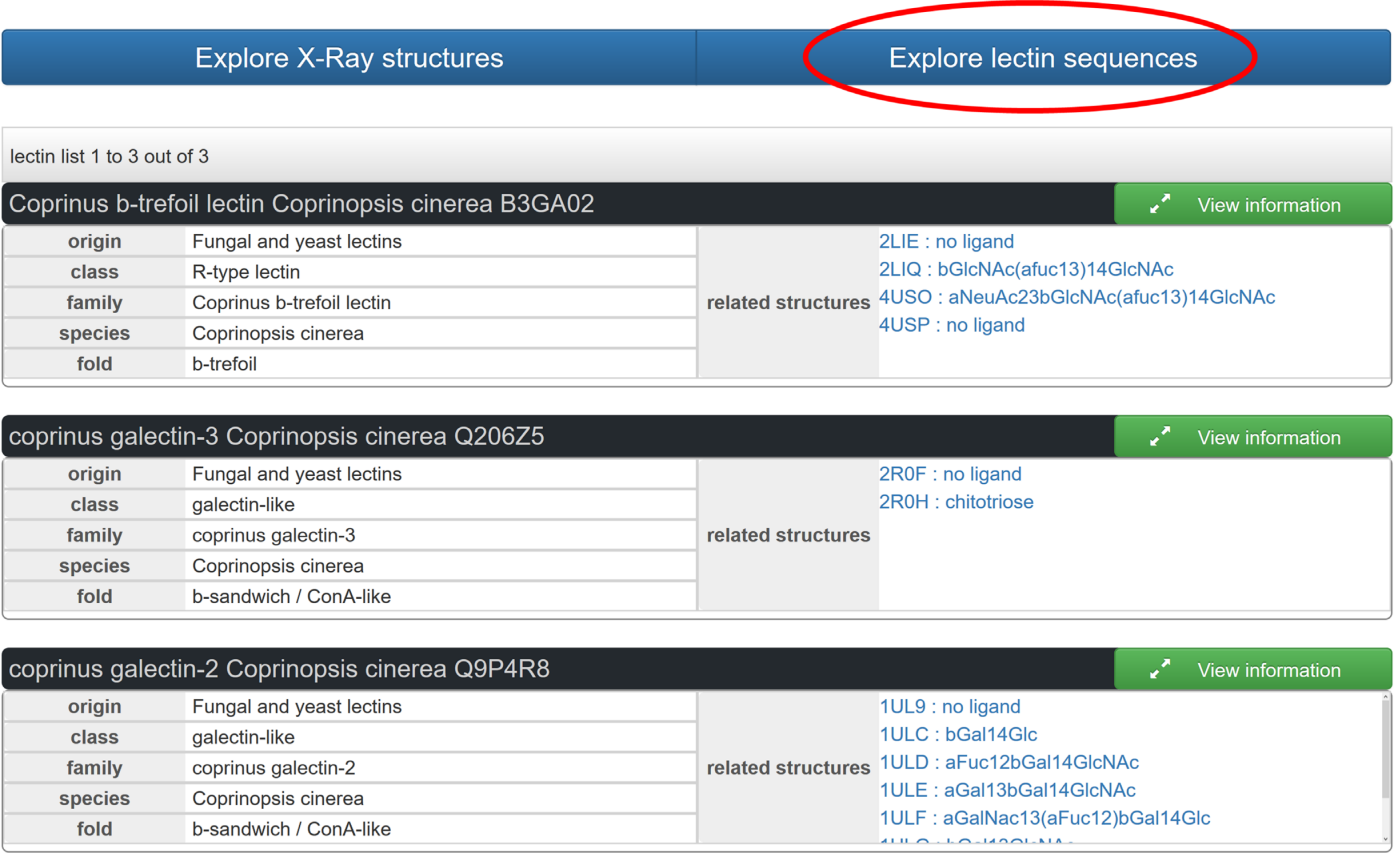
Full results from the search on lectin sequences present in the database, using ‘*Coprinopsis cinerea’* in the species field. More information can be obtained on each lectin by clicking the green button. All related structures (list in blue) can be clicked for opening the structure viewer.

UniLectin3D allows a precise taxonomic search for all lectins that have been structurally characterized in a given organism. For example, mushrooms contain a large variety of lectins that play a role in the protection against predators/feeders as well as in the establishment of symbiosis with plants (26). Searching for the lectins from the edible mushroom *Coprinopsis cinerea* can be performed by entering the species name and requesting ‘explore lectin sequences’. Three hits are returned and displayed in a graphical form (Figure 3). The search results indicate that two fungal galectins and one β-trefoil protein have been fully characterized in this organism and that 16 crystal structures have been described. Expanding the window related to Coprinus galectin-2 (Q9P4R8) using the ‘View information’ button opens a window showing all ligand oligosaccharides along with the information on crystal structures, literature and sequence (27) (see Figure S1 in Supplementary Data).

**Figure 4.**
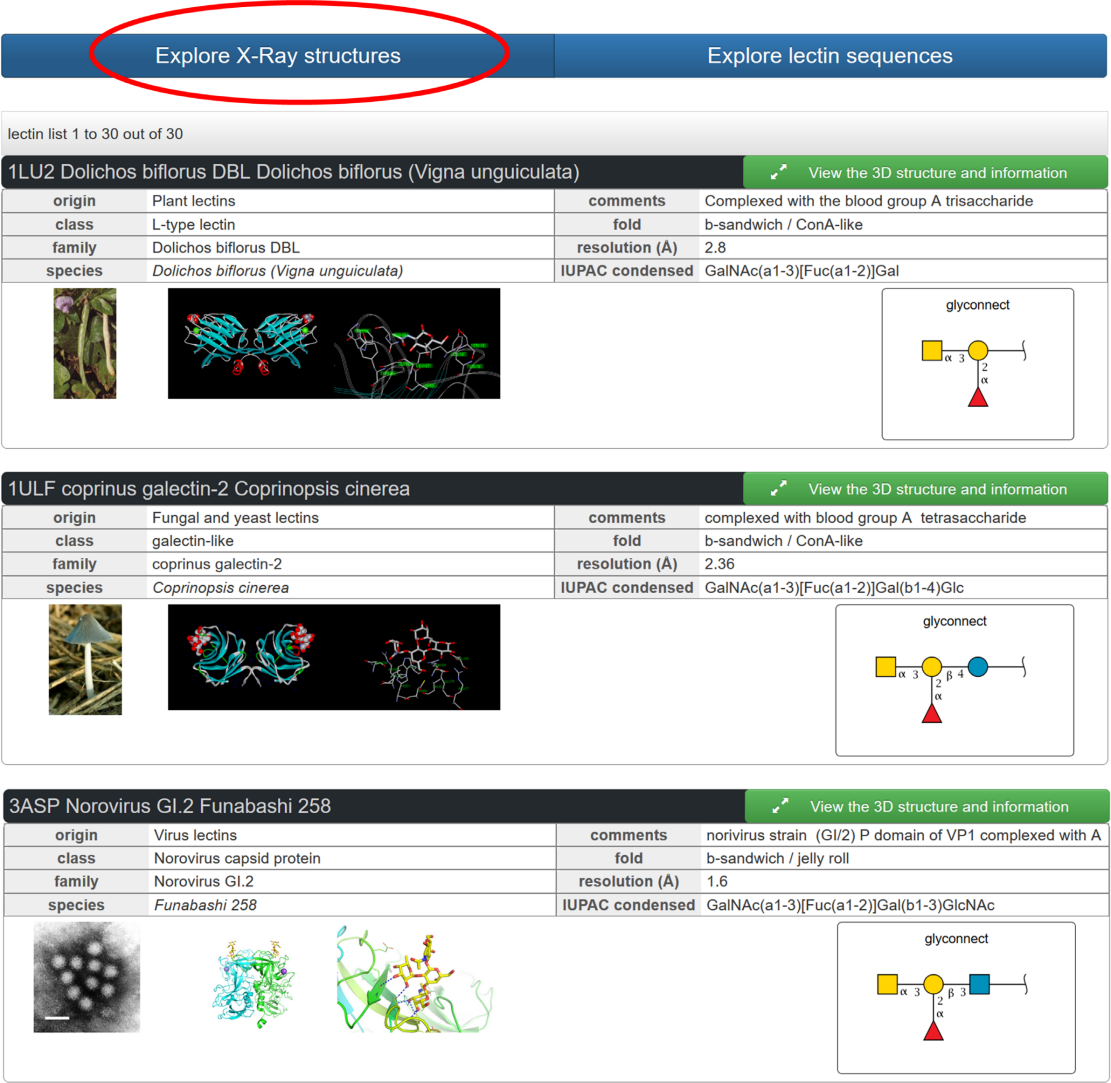
Partial results of the search on X-ray structures using the term ‘GalNAc(a1-3)[Fuc(a1-2)]Gal‘ (blood group A epitope) in the ‘IUPAC condensed’ window search. Three entries among the 31 obtained have been selected for display. More information can be obtained on each structure by clicking the green button. Clicking the ‘glyconnect’ button associated with the graphical representation of the glycan opens a database with sequence/function information on related glycoconjugates.

Oligosaccharide motifs that are complexed within the binding sites can also be searched. The well-documented human blood group epitopes that belong to the ABO and Lewis systems (28) are of special interest. These glycan epitopes are present at the surface of red cells, but also in mucins of airways and of gastrointestinal tracts where they can be recognized by a number of pathogens. Coevolution between hosts and pathogens resulted in a variety of blood group epitopes mirrored by a variety of protein receptors (29). The query for all lectin structures complexed with the blood group A epitope, i.e. search with the motif GalNAc(a1-3)[Fuc(a1-2)]Gal, outputs 31 3D-structures (Figure 4), corresponding to 13 different glycan receptors.

The detailed list of receptors is displayed in Supplementary Data (Table S1). The importance of blood group epitopes in infection is outlined by the prevalence of lectins from pathogens that represent 23 out of the 31 identified structures. Intestinal pathogens are highly represented with capsids and spikes from viruses responsible for gastroenteritis (norovirus and rotavirus), toxins from enteropathogenic bacteria (*Escherichia coli* and *Vibrio cholera*) and adhesins from stomach ulcer-causing *Helicobacter pylori*. As demonstrated by structure–function studies (30), the affinities of these lectins for human tissues vary as a function of the exposed blood groups oligosaccharide resulting in different epidemiology depending on the population phenotype (31). The airborne fungal pathogen *Aspergillus fumigatus* produces a lectin that attaches to fucosylated oligosaccharides in the lungs of immunocompromised patients (32). The remaining eight output structures are plant or fungal lectins that are of interest as potential biomarkers due to their ability to bind to human glycan epitopes.

## CURATED INFORMATION FOR EACH LECTIN

**Figure 5.**
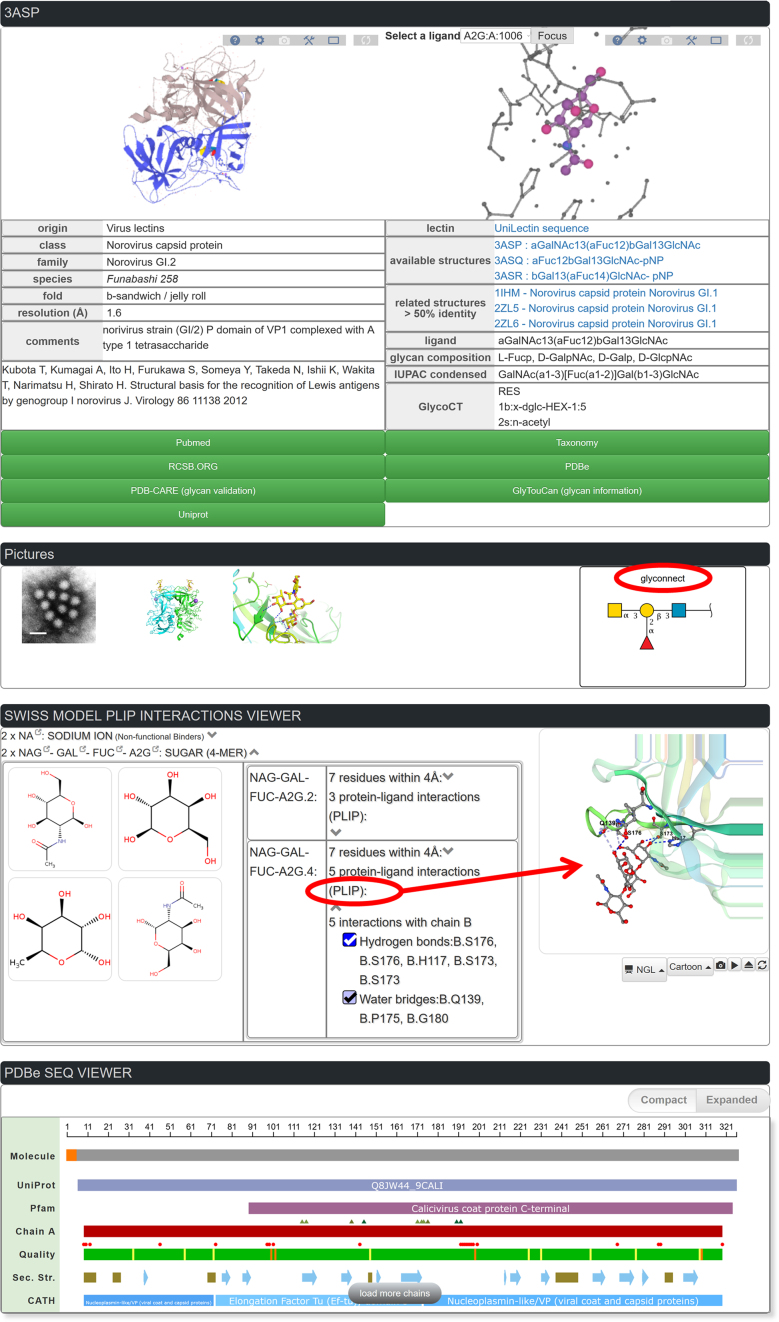
Information curated for one lectin structure together with the 3D structure viewer. The clickable related structures are listed in blue. Green buttons open external databases as well as the glyconnect button (circled in red). The graphical representation of non-covalent interaction for each ligand is obtained by selecting the monosaccharide of interest and clicking on the PLIP dropdown as indicated by red circle and arrow.

For each of the 1740 structures of the database, a feature viewer is available with different levels of information. The example of a norovirus capsid protein complexed with blood group A tetrasaccharide (PDB code: 3ASP) (33) is displayed in Figure 5. The top window displays the LiteMol viewer (34) for rapid visualization of the 3D X-ray structure. Options are available for full-screen display and for an interactive change of the display parameters. A second LiteMol viewer panel displays the specific ligand in its close interaction with the lectin-binding site. Curated classification, species, lectin fold, bibliographic references, resolution of the structure and complementary information in the form of comments (mutation,complex …) are also provided. Related structures with the same UniProt accession number or with strong sequence similarity (> 50%) are listed. Several levels of sequence information are provided for the glycan ligand, including the monosaccharide composition, the IUPAC nomenclature detailing linkages and the glycoCT format that is used in glycoinformatics for providing a unique description of glycan structure (35). Links to external databases (see Table 1) complement information regarding published articles, taxonomy, crystallography (36), sequence and glycan array specificity when available at the Consortium for Functional Glycomics site. Links to SugarBind, the database of pathogen lectins (23), are provided when relevant. Graphical representations of the lectin and binding site structures have been prepared using the PyMol (https://pymol.org/2/ by Schrödinger) or LiteMol (https://www.litemol.org/) (33) software. We take particular care in complying with the recommended standard representation of carbohydrates known as SNFG (37), and as mentioned above using the popular glycoCT encoding format (35). These options facilitate cross-linking to glyco-related external databases such as GlyConnect.

The third panel is devoted to the description of the molecular interactions between a lectin and its glycan ligand. In this process of molecular recognition, the formation of specific non-covalent interactions between proteins and ligands is the key factor (38). The PLIP is a tool to detect biologically relevant interactions, considering eight different interaction types and including water-mediated contacts (24). For this web server, PLIP interaction data were precalculated for all lectin–glycan complexes in the database and can be readily visualized in the browser using the PLIP applet from the SWISS-MODEL web server (39), based on NGL (40). Both a tabular format and a 3D applet let the user explore and toggle different interaction types for all binding sites in a lectin protein. We believe that this additional level of information not only will provide insights on lectin specificity but can also guide studies in the field of lectin engineering (8) and design of biologically active glycomimetics (7).

The last panel summarizes the information extracted from PDBe (41). It contains information about the lectin sequence with a 2D view of the location of the binding sites. All details of the residue-level mapping to UniProt, sequence families (Pfam), structure domains (SCOP, CATH), mutations, binding-site residues, structure quality and secondary structure are also listed. By default, the sequence–feature view shows the chain that has the maximum number of observed residues.

## DISCUSSION AND CONCLUSION

The lack of annotation of carbohydrate binding-proteins is a limitation to the functional interpretation of many cellular processes. Lectins are still poorly annotated in databases and genomes, therefore our effort in establishing UniLectin3D focuses on addressing this problem. The inclusion of cross-references to UniLectin3D in UniProt demonstrates that our data curation effort is heading in the right direction. Furthermore, a deeper embedding of UniLectin3D in the set of glyco-resources of the Glycomics@ExPASy collection is in preparation. Not only will SugarBindDB and GlyConnect shortly refer back to UniLectin3D (straightforwardly via their shared UniProt accession numbers) but we will also integrate lectin data in the glyco-epitope search tools (42). Currently, GlyConnect can be queried to reveal associations between glycosylated proteins and full glycan structures from which potential glyco-epitopes can be extracted. The next step of integration with UniLectin3D will open the possibility of displaying the potential set of lectins that bind those extracted epitopes.

Cross-linking with glycan ligand information is more complicated than with protein information since there is no equivalent yet of the UniProt universal resource in glycobiology. Furthermore, there is not yet either a distinction between annotated and unannotated glycans in glyco-databases. The GlyTouCan repository only registers glycan structures but do no annotate them (21), whereas the GlyConnect database annotates glycans but mainly stores those that are attached to proteins, leaving out glycolipids among others. Nonetheless, the international community of glycoinformatics in which we partake, is jointly addressing these issues and fast progress is expected to reduce the current difficulties of cross-referencing adequate glycan ligand information.

The amount and the quality of the 3D-structures of lectins from diverse origins are such that meaningful searches can be conducted leading to eventual new classifications, predictions of the occurrence of structural motifs (folds, quaternary structures, topology…) all the ways to understanding the structural basis underlying the exquisite binding to complex glycans. Since recognition of glycan is the main function of lectins, a particular effort was invested in the description and representation of interaction in the binding sites, with several types of image and graphical viewer. The use of PLIP (24) provides a detailed description of non-covalent contacts that represents a new resource for medicinal chemists involved in developing glycomimetics targeted to lectins.

The UniLectin project, which includes the Unilectin3D database, will expand in the next years with the integration of additional predictive tools. In particular, we aim at the automatic identification and annotation of lectins in newly sequenced genomes. This is a challenging task because the basis for the prediction of carbohydrate specificity or oligomerization state is not yet fully elucidated. As a consequence, the extraction of relevant information from sequences and multiple alignments to build specific models is not straightforward and requires substantial manual curation. We plan to tackle these issues within the UniLectin project by 2020.

## Supplementary Material

Supplementary Data
