# Supplementary material for: UniLectin3D, a database of carbohydrate binding proteins with curated information on 3D structures and interacting ligands
Source: Nucleic Acids Res. 2018 Sep 17;47(Database issue):D1236–44. doi: 10.1093/nar/gky832 (PMC6323968; doi:10.1093/nar/gky832)
Supplement: Supplementary Data [file gky832_supplemental_files.pdf]

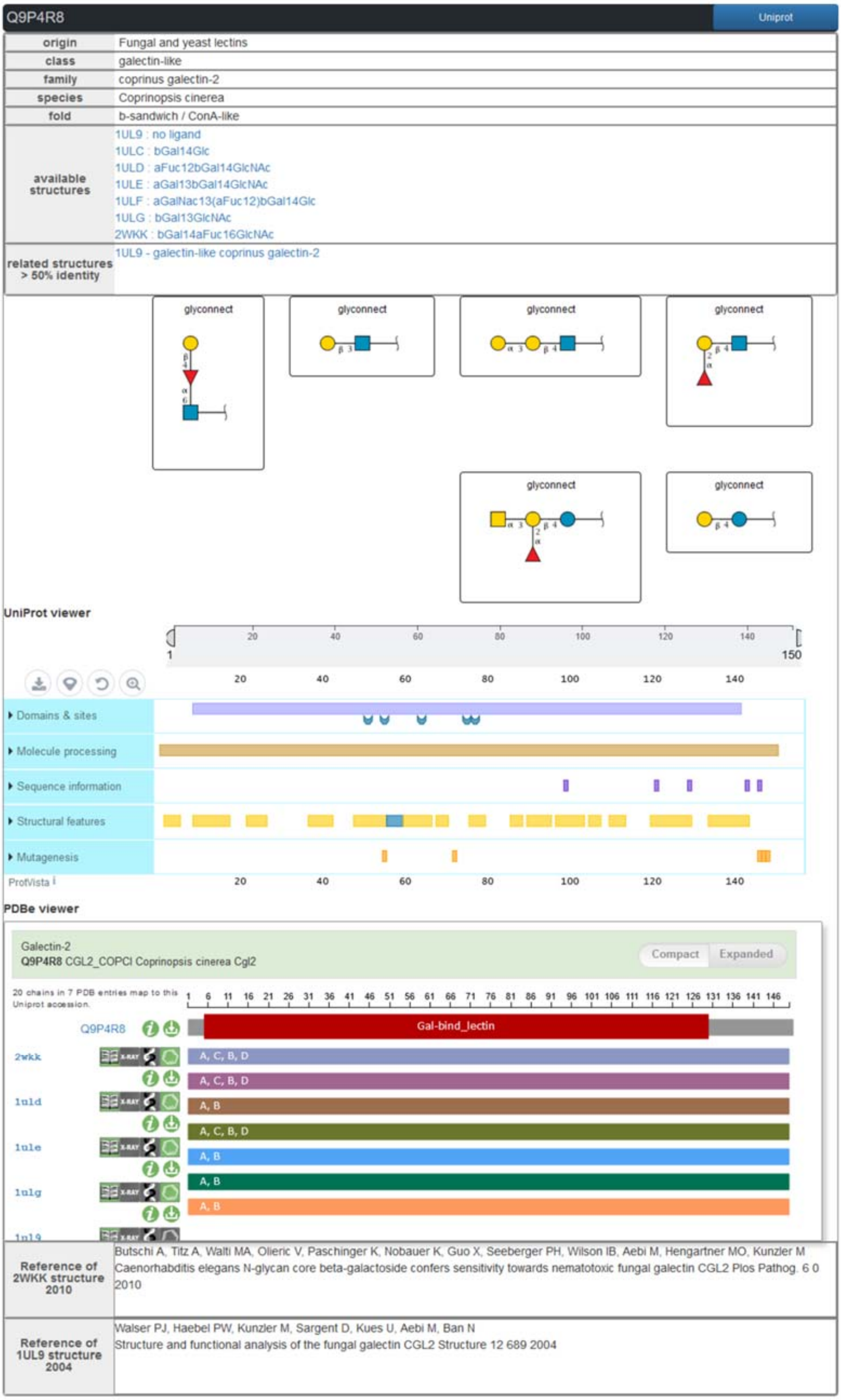

Figure 1S. Information obtained when clicking on the “View information” button for sequence Q9P4R8 (Galectin 2 from *Coprinopsis cinerea*). All crystal structures are listed, as well as all glycan ligands in lectin binding sites. Information is also available for the peptide chains present in each crystal structure.

Table 1S : Details of structures obtained in UniLectin3D after a search for lectins complexed with human blood group A epitope, i.e. ligand GalNAc(a1-3)[Fuc(a1-2)]Gal.

Lectin	Origin	Comment	PDB codes	ref
Capsid P-domain	Norovirus	Strains GI.1, GI.2, G1.7, GII.4, GII.10	2OBS, 2ZL7, 3ASP, 3D26, 3PA1, 3SLD, 4P26, 4WZT, 4X07	(1-8)
VP8* domain od spike protein	Rotavirus	Strains P[2], P[9], P[14]	4DRV, 4DS0, 5CB7, 5ZHO	(9-11)
Cholera toxin	*Vibrio cholerae*	AB_5 toxin	5ELD, 5ELE, 5ELF	(12)
Hybrid toxin	*Vibrio cholerae*	AB_5 toxin	3EFX	(13)
Heat-labile enterotoxin	enterotoxigenic *Escherichia coli*	AB_5 toxin	2O2L	(14)
FedF adhesin	enterotoxigenic *Escherichia coli*	F18 pili adhesin	4B4Q	(15)
BabA adhesin	*Helicobacter pylori*	HOP-OMP adhesin	5F7N, 5F7Y, 5F94	(16)
AFL	*Aspergillus fumigatus*	b-propeller lectin	4H4	(17)
ACG	*Agrocybe aegerita*	Fungal galectin	3WG3, 3WG4, 5XFD	(18,19)
CGL2	*Coprinopsis cinerea*	Fungal galectin	1ULF	(20)
BFL	*Bauhinia forficata*	Legume lectin	5T54	(21)
DBL	*Dolichos biflorus*	Legume lectin	1LU2 and model	(22,23)
WBA-I	*Psophocarpus tetragonolobus*	Legume lectin	2E7T	(24)

1. Cao, S., Lou, Z., Tan, M., Chen, Y., Liu, Y., Zhang, Z., Zhang, X.C., Jiang, X., Li, X. and Rao, Z. (2007) Structural basis for the recognition of blood group trisaccharides by norovirus. *J. Virology*, **81**, 5949-5957.
2. Choi, J.M., Hutson, A.M., Estes, M.K. and Prasad, B.V. (2008) Atomic resolution structural characterization of recognition of histo-blood group antigens by Norwalk virus. *Proc Natl Acad Sci U S A*, **105**, 9175-9180.
3. Kubota, T., Kumagai, A., Ito, H., Furukawa, S., Someya, Y., Takeda, N., Ishii, K., Wakita, T., Narimatsu, H. and Shirato, H. (2012) Structural basis for the recognition of Lewis antigens by genogroup I norovirus. *J Virol*, **86**, 11138-11150.
4. Bu, W., Mamedova, A., Tan, M., Xia, M., Jiang, X. and Hegde, R.S. (2008) Structural basis for the receptor binding specificity of Norwalk virus. *J Virol*, **82**, 5340-5347.
5. Hansman, G.S., Shahzad-Ul-Hussan, S., McLellan, J.S., Chuang, G.Y., Georgiev, I., Shimoike, T., Katayama, K., Bewley, C.A. and Kwong, P.D. (2012) Structural basis for norovirus inhibition and fucose mimicry by citrate. *J Virol*, **86**, 284-292.
6. Shanker, S., Choi, J.M., Sankaran, B., Atmar, R.L., Estes, M.K. and Prasad, B.V. (2011) Structural analysis of histo-blood group antigen binding specificity in a norovirus GII.4 epidemic variant: implications for epochal evolution. *J Virol*, **85**, 8635-8645.
7. Singh, B.K., Leuthold, M.M. and Hansman, G.S. (2015) Human noroviruses' fondness for histo-blood group antigens. *J Virol*, **89**, 2024-2040.

8. Shanker, S., Czako, R., Sankaran, B., Atmar, R.L., Estes, M.K. and Prasad, B.V. (2014) Structural analysis of determinants of histo-blood group antigen binding specificity in genogroup I noroviruses. *J Virol*, **88**, 6168-6180.
9. Hu, L., Crawford, S.E., Czako, R., Cortes-Penfield, N.W., Smith, D.F., Le Pendu, J., Estes, M.K. and Prasad, B.V. (2012) Cell attachment protein VP8* of a human rotavirus specifically interacts with A-type histo-blood group antigen. *Nature*, **485**, 256-259.
10. Yu, X., Mishra, R., Holloway, G., von Itzstein, M., Coulson, B.S. and Blanchard, H. (2015) Substantial Receptor-induced Structural Rearrangement of Rotavirus VP8*: Potential Implications for Cross-Species Infection. *Chembiochem*, **16**, 2176-2181.
11. Sun, X., Wang, L., Qi, J., Li, D., Wang, M., Cong, X., Peng, R., Chai, W., Zhang, Q., Wang, H. *et al.* (2018) Human Group C Rotavirus VP8*s Recognize Type A Histo-Blood Group Antigens as Ligands. *J Virol*, **92**.
12. Heggelund, J.E., Burschowsky, D., Bjornestad, V.A., Hodnik, V., Anderluh, G. and Krengel, U. (2016) High-Resolution Crystal Structures Elucidate the Molecular Basis of Cholera Blood Group Dependence. *PLoS Pathog*, **12**, e1005567.
13. Holmner, A., Lebens, M., Teneberg, S., Angstrom, J., Okvist, M. and Krengel, U. (2004) Novel binding site identified in a hybrid between cholera toxin and heat-labile enterotoxin: 1.9 A crystal structure reveals the details. *Structure*, **12**, 1655-1667.
14. Holmner, A., Askarieh, G., Okvist, M. and Krengel, U. (2007) Blood group antigen recognition by *Escherichia coli* heat-labile enterotoxin. *J. Mol. Biol.*, **371**, 754-764.
15. Moonens, K., Bouckaert, J., Coddens, A., Tran, T., Panjikar, S., De Kerpel, M., Cox, E., Remaut, H. and De Greve, H. (2012) Structural insight in histo-blood group binding by the F18 fimbrial adhesin FedF. *Mol Microbiol*, **86**, 82-95.
16. Moonens, K., Gideonsson, P., Subedi, S., Bugaytsova, J., Romao, E., Mendez, M., Norden, J., Fallah, M., Rakhimova, L., Shevtsova, A. *et al.* (2016) Structural Insights into Polymorphic ABO Glycan Binding by Helicobacter pylori. *Cell Host Microbe*, **19**, 55-66.
17. Houser, J., Komarek, J., Cioci, G., Varrot, A., Imberty, A. and Wimmerova, M. (2015) Structural insights into *Aspergillus fumigatus* lectin specificity - AFL binding sites are functionaly non-equivalent. *Acta Crystallographica*, **D71**, 442-453.
18. Kuwabara, N., Hu, D., Tateno, H., Makyio, H., Hirabayashi, J. and Kato, R. (2013) Conformational change of a unique sequence in a fungal galectin from Agrocybe cylindracea controls glycan ligand-binding specificity. *FEBS Lett*, **587**, 3620-3625.
19. Yamashita, K., Kuwabara, N., Nakane, T., Murai, T., Mizohata, E., Sugahara, M., Pan, D., Masuda, T., Suzuki, M., Sato, T. *et al.* (2017) Experimental phase determination with selenomethionine or mercury-derivatization in serial femtosecond crystallography. *IUCrJ*, **4**, 639-647.
20. Walser, P.J., Haebel, P.W., Künzler, M., Sargent, D., Kües, U., Aebi, M. and Ban, U. (2004) Structure and functional analysis of the fungal galectin CGL2. *Structure*, **12**, 689-702.
21. Lubkowski, J., Durbin, S.V., Silva, M.C., Farnsworth, D., Gildersleeve, J.C., Oliva, M.L. and Wlodawer, A. (2017) Structural analysis and unique molecular recognition properties of a Bauhinia forficata lectin that inhibits cancer cell growth. *FEBS J*, **284**, 429-450.
22. Casset, F., Peters, T., Etzler, M., Korchagina, E., Nifant'ev, N., Pérez, S. and Imberty, A. (1996) Conformational analysis of blood group A trisaccharide in solution and in the binding site of Dolichos biflorus lectin using transient and transferred nuclear Overhauser enhancement (NOE) and rotating-frame NOE experiments. *Eur. J. Biochem.*, **239**, 710-719.
23. Hamelryck, T.W., Loris, R., Bouckaert, J., Dao-Thi, M.-H., Strecker, G., Imberty, A., Fernandez, E., Wyns, L. and Etzler, M.E. (1999) Carbohydrate binding, quaternary structure and a novel hydrophobic binding site in two legume lectin oligomers from *Dolichos Biflorus*. *J. Mol. Biol.*, **286**, 1161-1177.
24. Kulkarni, K.A., Katiyar, S., Surolia, A., Vijayan, M. and Suguna, K. (2007) Generation of blood group specificity: new insights from structural studies on the complexes of A- and B-reactive saccharides with basic winged bean agglutinin. *Proteins*, **68**, 762-769.
